# Advanced Surface Modification for 3D-Printed Titanium Alloy Implant Interface Functionalization

**DOI:** 10.3389/fbioe.2022.850110

**Published:** 2022-03-01

**Authors:** Xiao Sheng, Ao Wang, Zhonghan Wang, He Liu, Jincheng Wang, Chen Li

**Affiliations:** ^1^ Department of Orthopedics, The Second Hospital of Jilin University, Changchun, China; ^2^ Orthopaedic Research Institute of Jilin Province, Changchun, China

**Keywords:** 3D-printed, titanium alloy, implant interface, surface modication, surface functionalization

## Abstract

With the development of three-dimensional (3D) printed technology, 3D printed alloy implants, especially titanium alloy, play a critical role in biomedical fields such as orthopedics and dentistry. However, untreated titanium alloy implants always possess a bioinert surface that prevents the interface osseointegration, which is necessary to perform surface modification to enhance its biological functions. In this article, we discuss the principles and processes of chemical, physical, and biological surface modification technologies on 3D printed titanium alloy implants in detail. Furthermore, the challenges on antibacterial, osteogenesis, and mechanical properties of 3D-printed titanium alloy implants by surface modification are summarized. Future research studies, including the combination of multiple modification technologies or the coordination of the structure and composition of the composite coating are also present. This review provides leading-edge functionalization strategies of the 3D printed titanium alloy implants.

## Introduction

**FIGURE 1:**
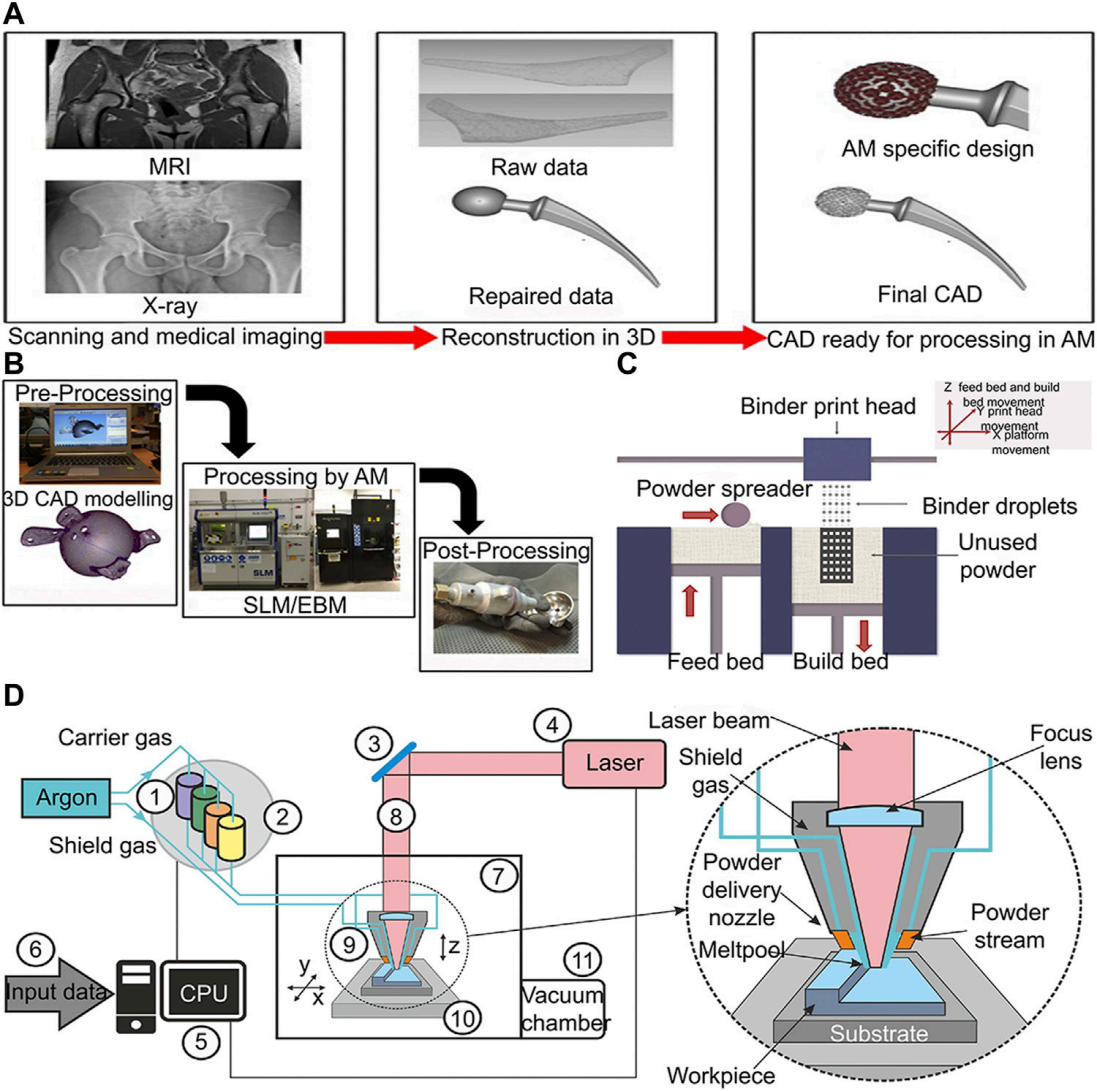
3D-printing of titanium alloy process diagram **(A)** 3D-printed customized implant data acquisition process (Sing et al., 2016). **(B)** Process chain for preparing orthopedic implants by 3D-printed technology (Sing et al., 2016). **(C)** Schematic diagram of powder bed process (Wang et al., 2017). **(D)** Lens process schematic diagram (Antolak-Dudka et al., 2019).

Titanium alloy has been extensively used in the medical fields of orthopedics, dentistry, and vascular surgery owing to its high strength, low density, high corrosion resistance, and excellent biocompatibility (Domínguez-Trujillo et al., 2018). Traditional titanium alloy implants have been manufactured by iso-material mold casting or subtractive technologies such as machining, multipoint forming and NC machining (Minto et al., 2020; Khorasani et al., 2016; Herzog and Tille 2021; Markopoulos et al., 2018). Hence, they are difficult to simulate the structure of cortical bone and cancellous bone in real bone tissue (Bozkurt and Karayel, 2021). With the advent of 3D-printed technology, these issues may be solved. The use of 3D-printed medical devices in the direct treatment of patients has increased considerably since 2015. And the most prevalent is in surgery, especially in orthopedics (36%) and orthopedic oncology (32%), followed by maxillofacial surgery (6%), neurosurgery (4%), and plastic surgery (1%) (Kermavnar et al., 2021). 3D-printed technology is divided into powder bed method and power deposit method. The energy source is controlled by a computer system to scan and treat discrete materials distributed layer by layer, in order to directly form parts with a 3D structure (Figure 1) (Wang et al., 2017). Various types of 3D-printed technologies such as fused deposit modelling (FDM), stereolithography, and laser sintering have been used in the manufacture of orthopedic implant (Liaw and Guvendiren, 2017). Compared to traditional implant manufacturing methods, 3D-printed technology has two advantages. In terms of microstructure, 3D-printed technology can precisely control the Young’s modulus of the prosthesis to match natural bone by predesigned pores that can effectively reduce the stress shielding effect of the implant and reduce the incidence of peri implant osteolysis (Minto et al., 2020). In terms of macro structure, implant shape can be designed with 3D-printed technology, accurately matching the complex bone defect through computed tomography (CT) images, so as to achieve the dual adaptation of the mechanical properties and shapes of the implant to the natural bone tissue (Wong, 2016). The different between traditional titanium alloy implant manufacturing method and 3D printing technology have been summarized in Table 1.

**TABLE 1:** Summary of different implant manufacturing technologies, manufacturing principle, cost and personalization.

Implant manufacturing technology	Manufacturing principle	Cost	Personalization	References
Machining	Using turning, milling and grinding ect. to form base material with specific shape	High	Poor	Khorasani et al. (2016)
Multipoint forming	Creating a mould that can be adapted to the part to be produced and thus it is not necessary to produce a mould for each individual part	Low	Poor	Herzog and Tille (2021)
NC machining	Using the control system sends out instructions to make the cutting tool make various movements that meet the requirements, and represent the shape and size of the workpiece in the form of numbers and letters for machining	High	Great	Markopoulos et al. (2018)
3D-printed technology	Firstly, the 3D image is obtained by CT scanning, then the raw material powder is deposited layer by layer by computer-controlled 3D printer, and finally the molten material is cast into a pre-designed 3D shape	Low	Great	Wang et al. (2017)

With the rising number of orthopedic implant surgeries, patients face the risk of bacterial infection, poor osseointegration, and aseptic inflammation (Bauer and Schils 1999; Ding et al., 2020; Zardi and Franceschi, 2020). Although titanium alloy has the advantages of excellent biocompatibility and corrosion resistance, titanium alloy has three obvious disadvantages (Domínguez-Trujillo et al., 2018). First, untreated titanium alloy implants always possess a bioinert surface that prevents reactions between the organism and implant. Second, a titanium alloy implant is unable to prevent bacterial infection by itself. Third, although titanium alloy materials have good biocompatibility, once the oxidation film on the surface has been damaged, harmful metal ions may enter the blood circulation, resulting in serious consequences (Wang C et al., 2018). In recent years, the development of surface modification technologies has provided new ideas for resolving the aforementioned problems. Researchers change the surface morphology or add certain substances to implant surface to achieve the effects of anti-infection, osteogenesis, wear resistance, corrosion resistance, and oxidation resistance (Zhang S et al., 2015; Li B et al., 2019; Maimaiti et al., 2020; Tang et al., 2020). Basically, 3D-printed titanium alloy surface modification technologies can be divided into three categories: chemical modification technologies, physical modification technologies, and biological modification technologies. Within chemical modification technologies, implants are usually exposed to a chemical solution or gas that bond to bioactive substances by chemical links (Zhang S et al., 2018; Llopis-Grimalt et al., 2020). Different from chemical methods, physical surface modification technologies do not change the chemical properties of substrate materials, but depend on lasers, high-energy particles, ultrasonics, and magnetic fields to modify the surface appearance and microscopic morphology of titanium alloy materials (Ma et al., 2017; Shin et al., 2017; Guo et al., 2020). Biological surface modification technologies may also form chemical bonds between implant surface and coating, but they mainly combine bioactive materials on the implant surface by using Van der Waals force, electrostatic interaction, and hydrogen bonds (Amin Yavari et al., 2020). The different surface modification technologies advantages and disadvantages have been summarized in Table 2. Some technologies often use multiple physical, chemical, and biological processes to establish various surface topographies on the implant surface.

**TABLE 2:** Summary of surface modification technology, advantages and disadvantages.

Surface modification technology	Advantages	Disadvantages	References
MAO	Low cost, simple equipment, easy operation, high coating efficiency, strong adhesion with base material, adapt to complex structures, high biological activity, high wear resistance	Reducing the fatigue strength of the base material	Fan et al. (2012), Wang et al. (2011), Yang Y. W et al. (2020), Myakinin et al. (2021), Nagarajan et al. (2018)
Anodizing	Low cost, simple equipment, easy operation, high biological activity, form TiO_2_ nanotube structures, reduce bacterial adhesion	Reducing the fatigue strength of the base material, the coating quality is poor than MAO	Tang et al. (2016), Minagar et al. (2013), Peng et al. (2013), Sarraf et al. (2021), Krzakala et al. (2013)
EPD	High-quality coatings, adapt to complex structures, precise controllable coating thickness	High temperature sintering is required when depositing specific materials	Pishbin et al. (2014), Seuss et al. (2014), Boccaccini et al. (2010), Narayanan et al. (2008)
CVD	High-quality coatings, adapt to complex structures	High cost, high equipment demand, high temperature environment required	Choy (2003), Zhao et al. (2019)
ALD	Precise thickness control, nano-precision coating, exceptional large-area uniformity, strong bonding strength, low growth temperature, excellent reproducibility	High cost, low working voltage, and slower coating production speed	Liu et al. (2017b), Bishal et al. (2015)
Alkali heating	Low cost, simple equipment, easy operation, high biological activity	The coating state cannot be accurately controlled	Tamilselvi et al. (2009), Kokubo and Yamaguchi (2010)
LSE	Accurately control the 3D shape of the coating	High temperature environment required	Fathi-Hafshejani et al. (2020), Shin et al. (2017)
AIP	Strong adhesion with base material, low deposition temperature	High equipment demand	Chung et al. (2011)
Shot peening	Increase fatigue resistance and wear resistance of implant	May have certain cytotoxicity	Liu et al. (2019)
UNSM	Improving the yield strength and wear resistance	High equipment demand	Karimbaev et al. (2020), Amanov et al. (2012)
LBL	Low cost, simple equipment, easy operation, precise coating control	Poor adhesion with base material	Zhang T et al. (2018), Ma et al. (2015)
Hydrogel	Low cost, simple equipment, easy operation adapt to complex structures, excellent drug carrier	Poor adhesion with base material	Yue et al. (2020)

**SCHEME 1:**
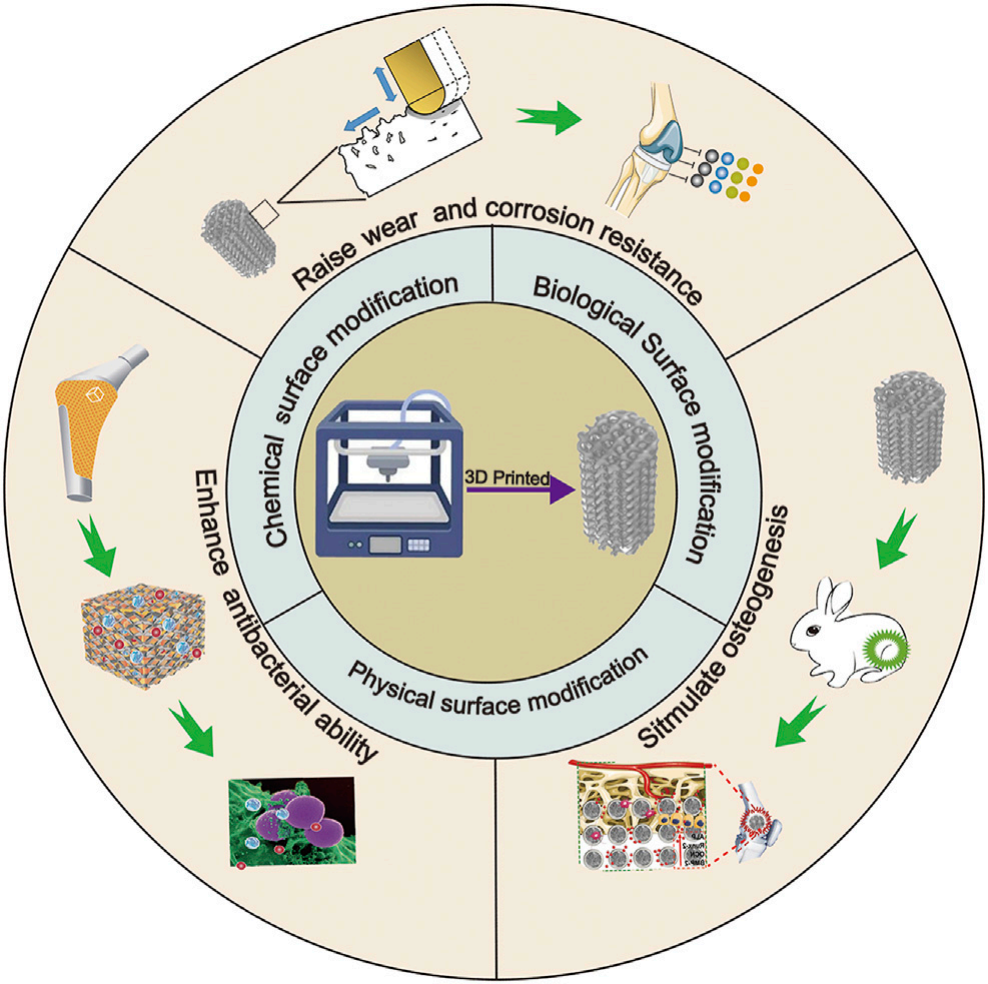
Surface modification technologies and biological functionalization of 3D-printed titanium alloy implants.

Surface modification technologies are key factors that enhance the bionic properties of implants. This review focuses on chemical, physical, and biological surface modification technologies in the application of 3D-printed implants. In addition, the article also summarizes the performance of implants after surface modification with respect to antibacterial, osteogenesis, and mechanical properties, and provides further details to improve the surface modification technologies and manufacture biomimetic implants with excellent performance in the future (Scheme 1).

## Surface Modification Technology

**FIGURE 2:**
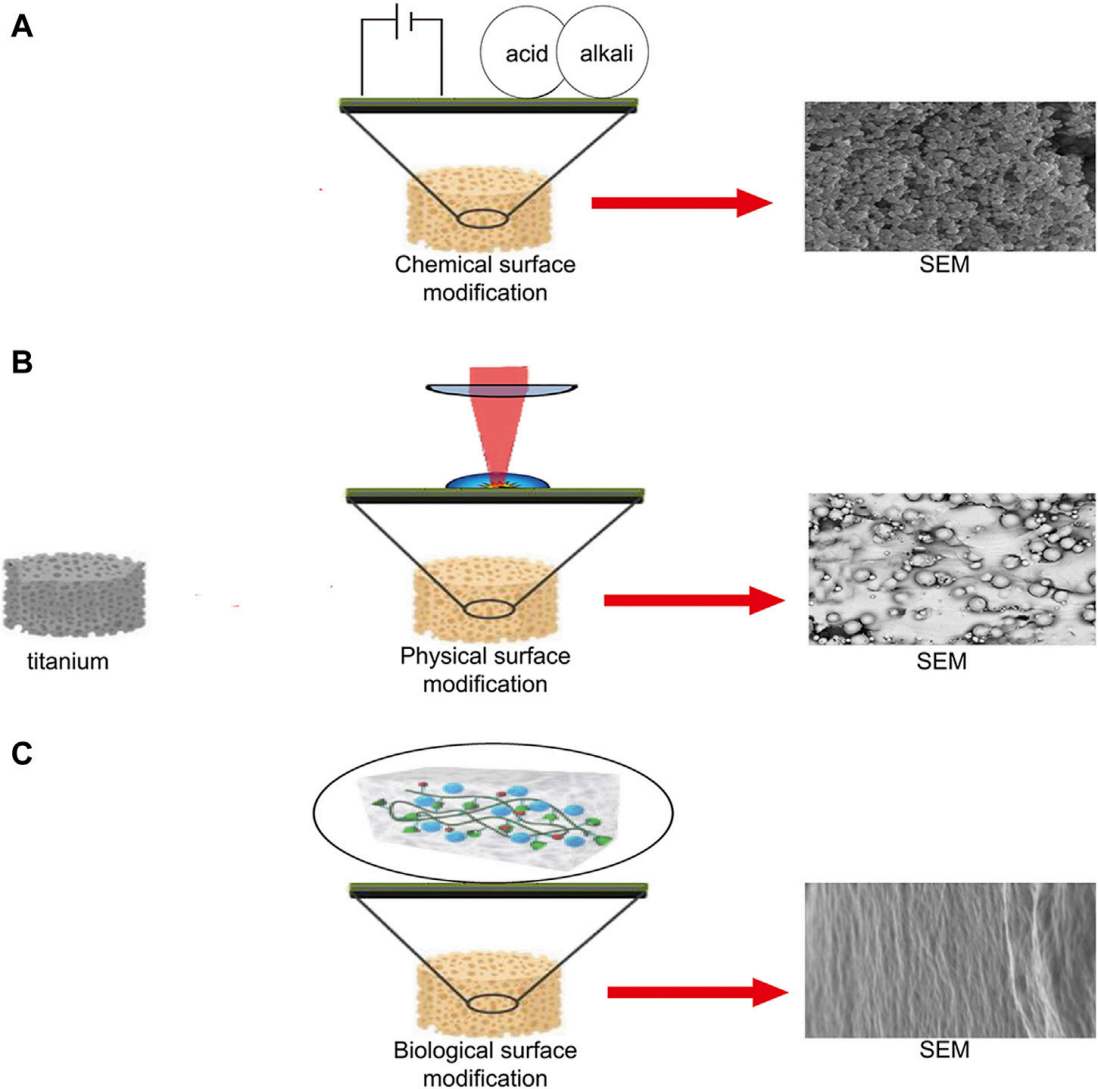
Schematic diagram of chemical, physical, and biological surface modification technologies. **(A)** Implant surface microstructure produced by electrophoretic deposition (Surmeneva et al., 2019). **(B)** Microstructure of implant surface produced by laser peening (Soyama and Takeo, 2020). **(C)** Surface microstructures of implants produced by hydrogel packaging (Mieszkowska et al., 2020).

Surface modification refers to the method of improving the biological properties by preparing coatings or changing the surface morphology of implants (Figure 2). The different surface modification technologies, coating materials and functions have been summarized in Table 3.

**TABLE 3:** Summary of surface modification technology, coating constructing methods, and functions.

Implantmaterial	3D-printed method			Surface modificationtechnology		Coating materials		Function	References
Ti_6_Al_4_V	SLM			MAO		Ag^+^, Sr, CaP, BMP-2, Vancomycin		MAO can produce micro-nano structures that promote osteogenesis on the implant surface, and different substances can be deposited by changing the electrolyte	Zhang W et al. (2020), Rodríguez-Contreras et al. (2020), Chen et al. (2019), Wei et al. (2020), Teng et al. (2019)
Ti_6_Al_4_V	SLM			Anodizing		Ag^+^, HA, MBG, MTANi		Anodic oxidation can form TiO_2_ nanotube structure on the surface of implants, which can be used as a carrier for a variety of drugs.	Shivaram et al. (2017), Qin et al. (2018), Zhao P et al. (2020), Li X et al. (2020)
Ti_6_Al_4_V	EBM			EPD		CaP, AgNPs, Vancomycin		Electrophoretic deposition can deposit solid particles suspended in solution on the implant surface to form a uniform coating	Bakhshandeh et al. (2017), Chudinova et al. (2019)
Ti_6_Al_4_V	EBM			CVD		Quercitrin		CVD deposited gaseous substances on the implant surface to form uniform coatings, and prepared coatings with different functions by depositing different substances	Llopis-Grimalt., et al. (2020)
Ti_6_Al_4_V	EBM			ALD		AlN		ALD can deposit materials on the implant surface in the form of monatomic films, and there is a correlation between each layer of atomic films.	Moll et al. (2021)
Ti_6_Al_4_V	SLM			Alkali heating		Sr, HA, Ga		Alkali heating can form micro-nano surfaces with osteogenic induction ability on the implant surface.	Shimizu et al. (2020), Rodríguez-Contreras et al. (2020), Li M et al. (2019)
Pure Ti, Ti_6_Al_4_V	SLA, LES, SLM Laser cladding			LSE		Si_3_N_4_, CaP, TiN-TiB, SiC		LSE can form a coating with 3D shape on the substrate by controlling the movement of high-energy laser source.	Das et al. (2014), Zanocco et al. (2020), Sahasrabudhe and Bandyopadhyay (2018), LiY et al. (2019)
Ti_6_Al_4_V	SLM			AIP		Mg, Ti-Cu/Ti-Cu-N		AIP forms a coating with strong adhesion on the implant surface by plasma bombarding gas substances.	Guo et al. (2020), Du et al. (2019)
Ti_6_Al_4_V	DMLS			Shot peening		No		Shot peening can produce a strengthening layer with high residual stress on the implant surface to improve the fatigue strength of the implant.	Soyama and Takeo (2020)
NiTi	SLM			UNSM		No		UNSM produces plastic deformation by impacting the implant surface with ultrasonic and shot peening to improve the wear resistance and tensile strength of the implant.	Ma et al. (2017)
Pure Ti	SLM			LBL		Gelatin, Chitosan, BMP-2, Vancomycin Rifampicin		LBL can alternatively deposit different substances on the implant surface by means of molecular interaction to form a coating with complete structure, stable performance, and specific function.	Amin Yavari et al. (2020), Jahanmard et al. (2020)
Ti_6_Al_4_V	EBM			Hydrogel		HA, Phloroglucinol, Simvastatin		Hydrogels can encapsulate different drugs in hydrogels and control the release of drugs through the degradation of hydrogels.	Zhang W et al. (2020), Kumar et al. (2018), Mieszkowska et al. (2020)

### Chemical Surface Modification

Chemical surface modification technologies include anodic oxidation, micro-arc oxidation, electrophoretic deposition, chemical vapor deposition, alkali heating and atomic layer deposition. These technologies facilitate the formation of chemical bonds to connect new substances with strong binding force. In addition, the chemical surface modification technologies have been adapted by the implants with complex shapes and have great prospects of application in the modification of 3D-printed implants (Xiu et al., 2016; Su 2021).

#### Anodizing

Anodizing belongs to electrochemical technology, which has been widely used in sewage treatment, battery energy, and bionanotechnology (Mandal et al., 2017; Davoodi et al., 2020; Janaina and Santos, 2021). In recent years, anodizing has been extensively used in implant surface for modifications owing to its simplicity, economy, and universality (Tang et al., 2016). In anodizing, titanium alloy is used as the anode, and lead or platinum plate is used as the cathode. The positive and negative ions in the electrolyte diffuse to the anode through the action of an electric field, and then the oxidation-reduction reaction occurs, forming a micro-nano structure on the titanium surface (Park et al., 2008). Anodizing is usually performed under galvanostatic conditions, up to a determined cell voltage or the passage of a determined charge, or by potential sweep. The process parameters which most frequently determine the properties of the growing oxide are the electrochemical parameters (current density, cell voltage, possible stabilization time) as well as the electrolyte specifications (com-position, pH, and temperature) and of course, the composition of the metal itself and its surface conditions (Diamanti, et al., 2011). With the increase in voltage, the size of the micropores of the oxide film increase, the anatase and rutile phases co-exist in the porous layer of titanium dioxide (TiO_2_) (Park et al., 2008). The TiO_2_ film with micro-nano structure exhibits lower contact angle (higher hydrophilicity) and higher surface energy (Minagar et al., 2013). That is conducive to the faster proliferation of osteoblasts on the implant surface. In addition, anodic oxidation also forms TiO_2_ nanotube structures on the titanium alloy. This nanotube structures can improve osseointegration by promoting the adhesion of the bone apatite layer. Furthermore, it also reduces bacterial adhesion to inhibit bacterial growth (Peng et al., 2013; Sarraf et al., 2021). Moreover, TiO_2_ nanotubes are often used as carriers to transform drugs or cells. On the one hand, researchers enhance bone growth by integrating CaP, BMP-2, metformin, and other osteogenic active substances into nanotubes (Li J. L et al., 2019; Hashemi et al., 2020; Yao et al., 2020). In addition nanotubes are used to incorporate antibacterial agents such as Ag^+^, Cu^2+^, vancomycin, and gentamicin to improve the antibacterial properties of implants (Caliskan et al., 2014; Li M et al., 2015; Wang et al., 2016; Zong et al., 2017). The diameter and length of TiO_2_ nanotubes are dependent on electrochemical oxidation conditions such as electrolyte type, concentration, pH, applied potential, and oxidation time (Minagar et al., 2012). However, there is no explicit data to determine the optimal nanotechnology that is conducive to cell response, and further research is needed to find the optimal size of nanotube length and diameter.

#### Microarc Oxidation Technology

Microarc oxidation (MAO), also known as plasma electrolytic oxidation (PEO), is a common metal surface modification technology (Shimabukuro, 2020). As an upgrade of anodic oxidation technology, MAO can prepare higher quality coatings. Compared with anodic oxidation, MAO coating has higher wear resistance and corrosion resistance (Chen et al., 2007). MAO is the method of using arc discharge to enhance and activate the reaction on the anode on the basis of ordinary anodic oxidation, to form a high-quality reinforced ceramic film on the metals (Li et al., 2004). In the electrolytic oxidation process, various parameters affect the quality of the coating, such as voltage, type of current, electrolyte composition, substrate material, and process duration (Sikdar et al., 2021). Similar to anodizing, MAO also forms porous micro-nano structure on the implant surface. The structure not only enables the implant to form contact bone formation with the surrounding bone tissue rather than distance bone formation, which greatly increases the binding force and stability of the implant, but also enhances the wear resistance of implant (Xiu et al., 2016). Moreover, the implant surface treated by MAO has high osteogenic activity. This may be because the micro-nano porous surface has a high degree of roughness and hydrophilia (Ribeiro et al., 2015). This is more conducive to the adhesion and proliferation of osteoblasts. Furthermore, MAO can also firmly binds to the materials on the surface of the implant by electrolysis of a specific electrolyte solution to prepare coatings (Komarova et al., 2020). Common coatings include CaP, Sr^2+^, BMP-2, chitosan, and vancomycin (Yao et al., 2010; Kim S.-Y. et al., 2019; Li N et al., 2019; Shimabukuro 2020; Zhang T et al., 2020; Zhao B et al., 2020). In addition, researchers are combining MAO with other surface modification technologies to produce multifunctional coatings. For instance, ultrasonic MAO (UMAO) was achieved by combining ultrasonic technology with MAO. A study showed that antibacterial properties and cell adhesion of Ti-Cu alloy can be enhanced by UMAO treatment (Hu et al., 2020). The microwave-assisted hydrothermal method is used to treat the nano-surface formed by MAO to form discrete nano-morphologies and retain the original micro pores, sub-micron pores, surface calcium, and phosphorus content. Lin et al. (2019) reported that MAO coating treated by MWDD has strong hydrophilicity and cell adhesion. Finally, the coating prepared by MAO may reduce the fatigue strength of implants and the fatigue limits of the MAO samples decreases as the coating thickness increasing (Kong et al., 2015). Therefore, in the future work, it is crucial to find out the best process parameters to control the thickness of MAO coating.

#### Electrophoretic Deposition Technology

Electrophoretic deposition (EPD) is used to prepare high-quality coatings on materials (Pishbin et al., 2014). Over the past few years, EPD has accelerated its application in the field of implant surface modification because of lower cost, simple process, and superior coating quality (Maciag et al., 2021). Unlike anodizing, anodizing is the anodic electrochemical technique while electrophoretic and cathodic depositions are the cathodic electrochemical techniques (Kim and Ramaswamy 2009). EPD is a colloidal treatment generally performed in a two-electrode electrolyte cell. Under the action of an electric field, the charged particles suspended in the solution move and are deposited on the substrate in an orderly manner, thus forming films and coatings (Boccaccini et al., 2010). The electric field and colloidal solution determine the properties of EPD coating. Among the most important parameters related to the EPD suspension, particle size is worth mentioning, as it directly affects the stability of the electrolyte. No general law has been defined regarding this criterion, although concerns with respect to the settlement due to gravity of larger particles have been reported (Bakhshandeh and Amin Yavari 2018). The advantage of EPD is prepares uniform coatings and achieves a precise controllable coating thickness (Seuss et al., 2014). Furthermore, owing to the electrolytic process being carried out in solution, EPD is capable of covering the substrate materials with a complex shape. EPD is widely used in preparing inorganic coatings on the surface of orthopedic implants, such as hydroxyapatite (HA), graphite oxide (GO), and Ag^+^ in coatings (Boccaccini et al., 2010; Shalom et al., 2018; Dulski et al., 2020; Srimaneepong et al., 2020). Moreover, the depositing environment of EPD is milder than the technologies requiring high temperature environment, such as plasma spraying and chemical vapor deposition. Accordingly, EPD is suitable for the preparation of organic bioactive coatings. For the past few years, EPD has been used to deposit various organic bioactive coatings such as bovine serum albumin (BSA) coating and double-layer silk fibroin (SF) coating layers (Hohn et al., 2017; Cheng et al., 2021). These biomimetic coatings show more powerful osteoinduction than traditional inorganic coatings.

#### Chemical Vapor Deposition Technology

Chemical vapor deposition (CVD) is used for solvent-free preparation of thin films and coatings (Khlyustova et al., 2020). The CVD system comprises three parts: a chemical gas precursor supply system, a chemical vapor repository reactor, and an exhaust gas treatment system. The CVD process begins with the generation of reactive gas reactants in the chemical gas pre-supply system; then, the reactants are pushed into the reactor with inert gas for reaction, and finally the reactants are adsorbed on the heated substrate to form a thin film. CVD coats the base materials with intricate shapes and deposits thin films with excellent coverage (Choy, 2003). CVD is often used to prepare diamond coatings (Pandey et al., 2021). For instance, Aaqil Rifai et al. (2018) prepared diamond coating on the surface SLM-Ti by CVD. The experimental results showed that the coating can promote cell proliferation and inhibit the growth of bacteria. However, complex equipment and high cost limit the application of CVD. In addition, traditional CVD may damage temperature-sensitive materials (base materials or coating materials). Moreover, the inefficiency of the precursor gas heating process leads to a major waste of energy (Zhao et al., 2019). Recently, researchers have transformed the energy supply mode of CVD by using different energy sources, so that CVD can be carried out at lower temperature, such as plasma enhanced chemical vapor deposition (PCVD) and initiation chemical vapor deposition (iCVD). In the PCVD process, high-energy plasma is used to provide the energy necessary for the reaction and render the preparation of coatings with a high temperature environment unnecessary (Okada, 2016). Moreover, iCVD is a new type of green polymer film preparation method. Its principle is to utilize an initiator to crack at a lower heating temperature to polymerize the monomer into a polymer film and deposit on the surface of the substrate (Su et al., 2020). The reaction conditions of iCVD are milder and more controllable than PCVD, and it is able to perfectly retain the required functional groups. In this regard, iCVD has the advantage of depositing organic coating materials. In a study, iCVD was used to secure rhBMP2 to the surface of titanium implants. Compared to the untreated implant, iCVD-treated implant significantly increases osteoinduction and calcium deposition (Youn et al., 2019).

#### Alkali Heating

Alkali heating is a simple and economical surface modification technology. It is often used to increase the surface of titanium alloys osteoconductivity as well as osteoinductivity (Kokubo and Yamaguchi 2015). In the process, the titanium alloy material is immersed in an NaOH aqueous solution at 60°C for 24 h, and then the sample is maintained at 600–800°C to obtain a porous oxide surface (Kim et al., 1996). The surface treated by the alkali heating can generate rutile and a feather-like sodium titanate structure, and induce a strong apatite formation ability in SBF (Tamilselvi et al., 2009; Kokubo and Yamaguchi 2010). That is because the sodium titanate on the surface of the Ti metal releases Na^+^ via exchange with the H_3_O^+^ in SBF so as to produce a local alkaline environment on the Ti metal. Consequently, the surface of the Ti metal heat-treated after exposure to the alkali solution is negatively charged. Its surface combine with the positively charged Ca^2+^, forming an amorphous calcium phosphate (Kokubo and Yamaguchi 2015). Furthermore, the surface treated by alkali heating presents a characteristic of a nano-double crystal state. The rough surface plays a pivotal role in increasing the contact between the bone and implant (Tsukimura et al., 2011). Alkali heating can be used as a pretreatment for other surface modification technologies and ensures the biological activity of the implant surface when preparing the implant surface with additional functions.

#### Atomic Layer Deposition

Atomic layer deposition (ALD) is an approach in which substances are deposited layer by layer on the substrate in the form of monolayer atom, and is used to prepare ultra-thin surface coatings (Listewnik et al., 2019). During ALD, the reactive gas is successively introduced into the reactor for reaction, and only one layer of atoms is deposited in each reaction; hence, each layer of atomic film is associated. This sequential process is a key difference between CVD and ALD, which consequently makes ALD a self-limiting reaction without gas phase reactions and gives unique characteristics to this method (Hashemi Astaneh et al., 2021). ALD has several obvious advantages such as precise thickness control, nano-precision coating, exceptional large-area uniformity, strong bonding strength, low growth temperature, excellent reproducibility, and resistance to sensitive substrates’ applicability (Liu H. et al., 2017). For 3D printing implants with complex porous structure, ALD can perfectly form a uniform film on its surface (Hashemi Astaneh et al., 2021). However, higher cost, lower working voltage, and slower coating production speed limit the application of ALD in the implant coating field (Bishal et al., 2015). To solve these problems, plasma-enhanced ALD (PEALD) and free radical-enhanced ALD (REALD) were proposed and established. PEALD can operate at lower temperatures than traditional thermal ALD, which is conducive to depositing unstable polymers at a high temperature. However, PEALD needs to provide the energy required for the reaction through plasma dissociation, which leads to damage of the substrate surface exposed to the environment of plasma discharge and high-energy electron bombardment (Li P et al., 2019). REALD is similar to PEALD in that, it exposes the cultured substrate and film to free radicals and eliminates substrate exposure to high-energy ions and electron bombardment. These free radicals, as reactants, are formed by gas dissociation using a hot wire feeder. Owing to the reactive free radicals, the process can be carried out at a lower temperature (closer to room temperature) (Bishal et al., 2015).

### Physical Surface Modification

Physical surface modification mainly changes the ultrastructure of the implant surface, including laser surface engineering, arc ion plating, shot peening, and ultrasonic nanotechnology. Physical modification is often used to enhance the wear resistance, corrosion resistance, and oxidation resistance. Compared to chemical modification, the bonding force of the coating is weak.

#### Laser Surface Engineering

Laser surface engineering (LSE) is a material processing method that can be utilized to manufacture implants and also to modify the surface of implants. When LSE modifies the implant surface, it only changes the texturing of the surface, and maintains the chemical state of the substrate materials (Kurella and Dahotre, 2005). During LSE, the deposited substances need to be first coated on the implant surface, and then melted by high-energy laser irradiation and combined with the implant surface (Fathi-Hafshejani et al., 2020). The advantage of LSE is that it can precisely control the texture of the coating, including the formation of the size and morphology of the pre-determined pores (Shin et al., 2017; Fathi-Hafshejani et al., 2020). Implant surface texture is a key for biomedical devices/surfaces because the materials-cells interaction is largely affected by surface morphology and their mechanical properties (Dong et al., 2021). One study reported that the laser micro/nanotexturing process modified the surface properties related to osseointegration, include biocompatibility, protein adsorption and cell/surface interactions (Shivakoti et al., 2021). Therefore, the design of the micro morphology of the implant surface may be able to promote the osteogenesis of the implant surface, inhibit the growth of bacteria and improve the wear resistance of the implant. In addition, LSE is well suitable for high temperature-resistant inorganic coating of materials (e.g., CaP, Si_3_N_4_, TiN-TiB, SiC) (Das et al., 2014; Sahasrabudhe and Bandyopadhyay 2018; Li P et al., 2019; Zanocco et al., 2020). Niobium (Nb) is a costly refractory material with greater wear resistance and biocompatibility (Zhang Y et al., 2015). A study reported that porous Nb coating can be effectively prepared on the surface of titanium alloy by SLM, demonstrating that SLM can prepare coatings with individually customized shapes and/or porosity from IVB and VB biomedical metals and their alloys (Zhang Y et al., 2015).

#### Other Physical Modification

Arc ion plating (AIP) is an excellent physical vapor deposition technology that can produce strong adhesion coatings at low temperature (<170°C) (Tsou et al., 2012; Liu L. et al., 2017). The AIP device is mainly composed of a deposition chamber, an arc power supply, a pumping system, and a gas flow control system. During the deposition process, the gas enters the deposition chamber and is bombarded by the metal ions emitted by the highly ionized arc onto the surface of the substrate, which then condenses on the substrate surface in the form of a solid film to form a coating (Chung et al., 2008). AIP has advantages over other technologies in the preparation of TiO_2_ coatings, such as high growth rate, strong film adhesion, and low deposition temperature (Chung et al., 2011). Finally, AIP is suitable for the preparation of inorganic metal coatings including MgCu, CaP, and TiCu/Ti-Cu-N coatings (Yu et al., 2018; Zhao et al., 2019; Guo et al., 2020).

Shot peening is an effective method to increase fatigue resistance and wear resistance of an implant (Liu et al., 2019). Shot peening can be divided into traditional mechanical shot peening and laser shock peening. In the mechanical shot peening process, a high-speed projectile stream is injected onto the surface of the material to cause plastic deformation. Then, residual stresses that are useful to improve the fatigue performance are introduced to the surface of the part (Nie et al., 2020). Laser shock peening compared to the mechanical shot peening is a non-contact process. This type of surface treatment can only be carried out using an intense laser pulse directed at a material surface in very short intervals rather than a continuous wave laser beam. The facilitated compressive residual stress is as much as four times larger than that of the mechanical shot peening technique (Shukla et al., 2013). The ability of shot peening to improve fatigue strength is suitable for the application of 3D-printed titanium alloy implants with weak fatigue strength. However, biocompatibility, osseointegration, and cytotoxicity of the shot-peened implants need to be further investigated.

Ultrasonic nano-crystal surface modification (UNSM) is a severe plastic surface deformation technology that can enhance the overall performance of metallic materials, in particular, the yield strength (Karimbaev et al., 2020). UNSM introduces plastic deformation, which leads to refinement of the grain size and high residual compressive stress on the surface and subsurface layers, while improving the mechanical properties of metal materials (Karimbaev et al., 2020). UNSM used the superposition of ultrasonic low frequency vibration on the static load to generated severe plastic deformation on the material surface, which further causes to surface nanocrystallization. The plastic deformation produced on the material surface during the UNSM process was induced by mechanical impacts. The high-frequency impact of the ball causes severe plastic deformation on the material surface, which leads to the introduction of high-density dislocation and grain boundaries. Thus, the nanostructured layer with a certain gradient is received (Amanov et al., 2012). The UNSM technique is a cold-forging process in which the ball made of silicon nitride and tungsten carbide mechanically impact on the material surface at a constant vibration frequency of 20 kHz and a certain region is processed within a certain period of time. Researchers can control the nanostructure surface layer thickness, mechanical properties, tribological properties and fatigue properties by changing the impact parameters (Liu et al., 2021). UNSM before the use of 3D printed implants can effectively prolong their service life, which is of great significance to patients who need joint replacement surgery.

### Biological Surface Modification

Biological surface modification is a primary technology that combines organic bioactive materials such as proteins on the surface of implants through electrostatic interaction, hydrogen bonds, and other forms. Different from chemical modification, it does not involve complex chemical reactions. Biological surface modification can deposit most organic biological coating materials. Therefore, biological modification has gradually evolved into an extensively researched topic for implant surface modification.

#### Layer-By-Layer Self-Assembly

**FIGURE 3:**
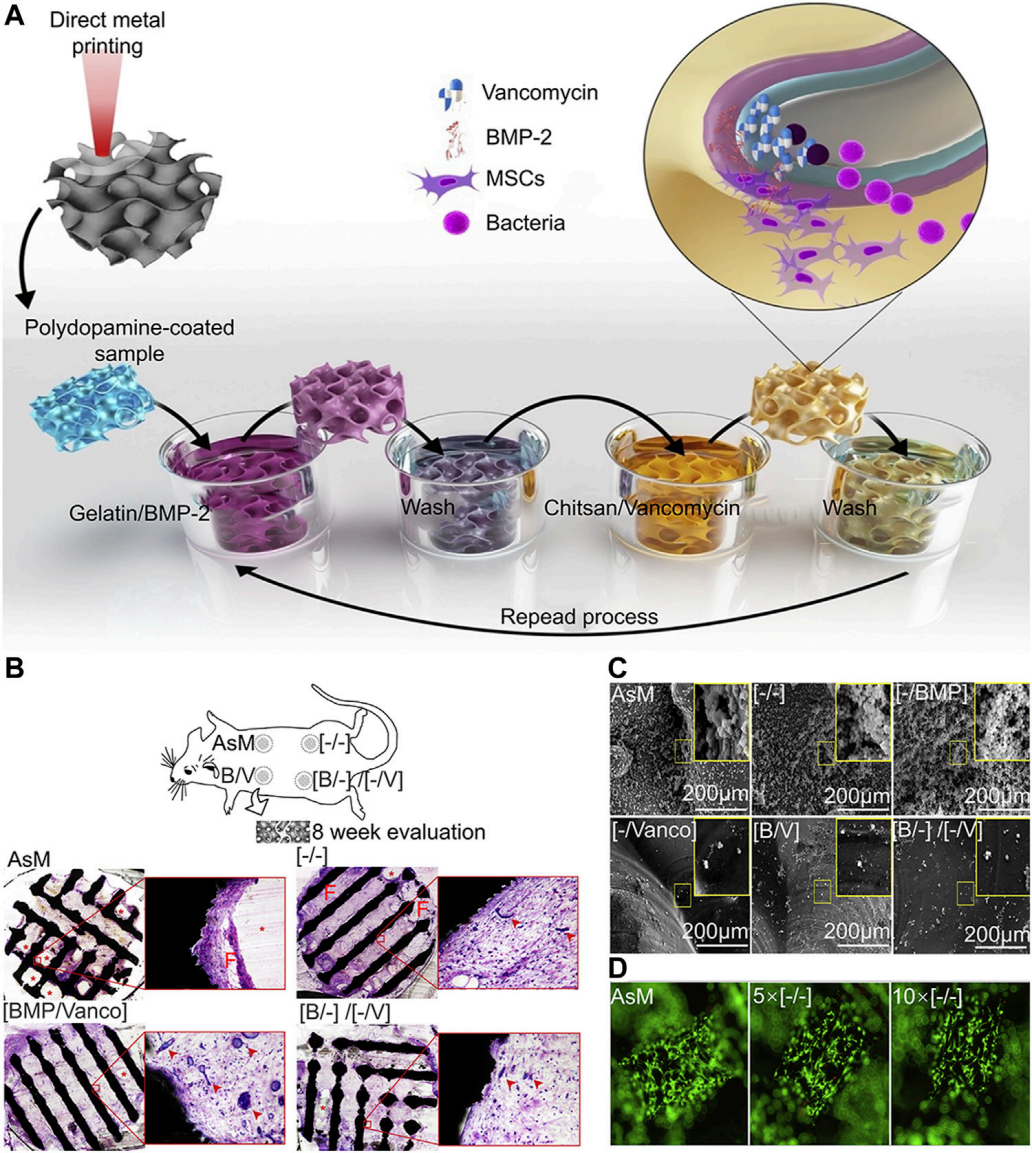
LBL vancomycin and BMP-2-coated implants (Amin Yavari et al., 2020). **(A)** Schematic illustration of the layer-by-layer coating process. **(B)** The biocompatibility of the scaffold was analyzed 8 weeks after implantation, the porous Ti structures did not induce an adverse tissue response in any of the groups, shown by the absence of acute inflammation or fibrous encapsulation at the material-tissue interface. In the case of any LBL remnants, no acute inflammatory response was seen around the polymer. At the same time, we observed a high density of blood vessel formation. **(C)** Representative images of planktonic and adherent bacteria on the surfaces of different experimental groups. In the experimental group containing vancomycin, the number of bacteria was significantly less. **(D)** In the live death staining experiment, the surface of the implant was completely covered by living cells, and the surface coating had no inhibitory effect on cell adhesion and proliferation.

Layer-by-layer (LBL) self-assembly is a film preparation method that has been widely used in various biomedical applications (Zhang T et al., 2018). The principle of LBL is to develop a laminated coating by alternating deposition of opposite-load polyelectrolytes on the surface of a laden substrate (Escobar et al., 2020). In the LBL process, multilayer films are deposited on the substrate surface through alternating adsorption of interactive materials, including polyelectrolytes, micelles, GO, antiparticles, and proteins (Park et al., 2018; Alkekhia et al., 2020). Hydrophobicity, Van der Waals forces, hydrogen bonds, covalent bonds, and bio-specific interactions promote film growth and are the main driving force of assembly (Park et al., 2018; Alkekhia et al., 2020). In addition, LBL can fabricate controllable coatings on almost all materials (Ma et al., 2015). For instance, fine control of composition, thickness, and topography can be achieved by adjusting the assembly parameters involving solution properties, like concentration, ionic strength, and pH, and process parameters, such as temperature, time, and drying conditions. Compared to other methods for fabricating nanofilms, there are three prominent advantages of the LBL, these include precise control of the composition and structure of nanofilms, large-scale fabrication capacity on various types of substrates regardless of size and shape, and mild and confined formation environments (Zhang T et al., 2018). Furthermore, LBL is good at manufacturing thin films for drug delivery, because it has no restriction on the size or shape of the substrate and can avoid the inactivation of some active substances caused by exposing the coating material to high temperature or high pressure (Park et al., 2018). Owing to the multivalent interaction between multilayer film and film components, it can carry high drug loads. Changing the number of layers can adjust the dose of the loaded drug (Alkekhia et al., 2020). In general, LBL is capable of achieving the loading and release of various types of drugs on the implant, thereby achieving the goal of biofunctionalization of the implant (Figure 3).

#### Hydrogel Combination

**FIGURE 4:**
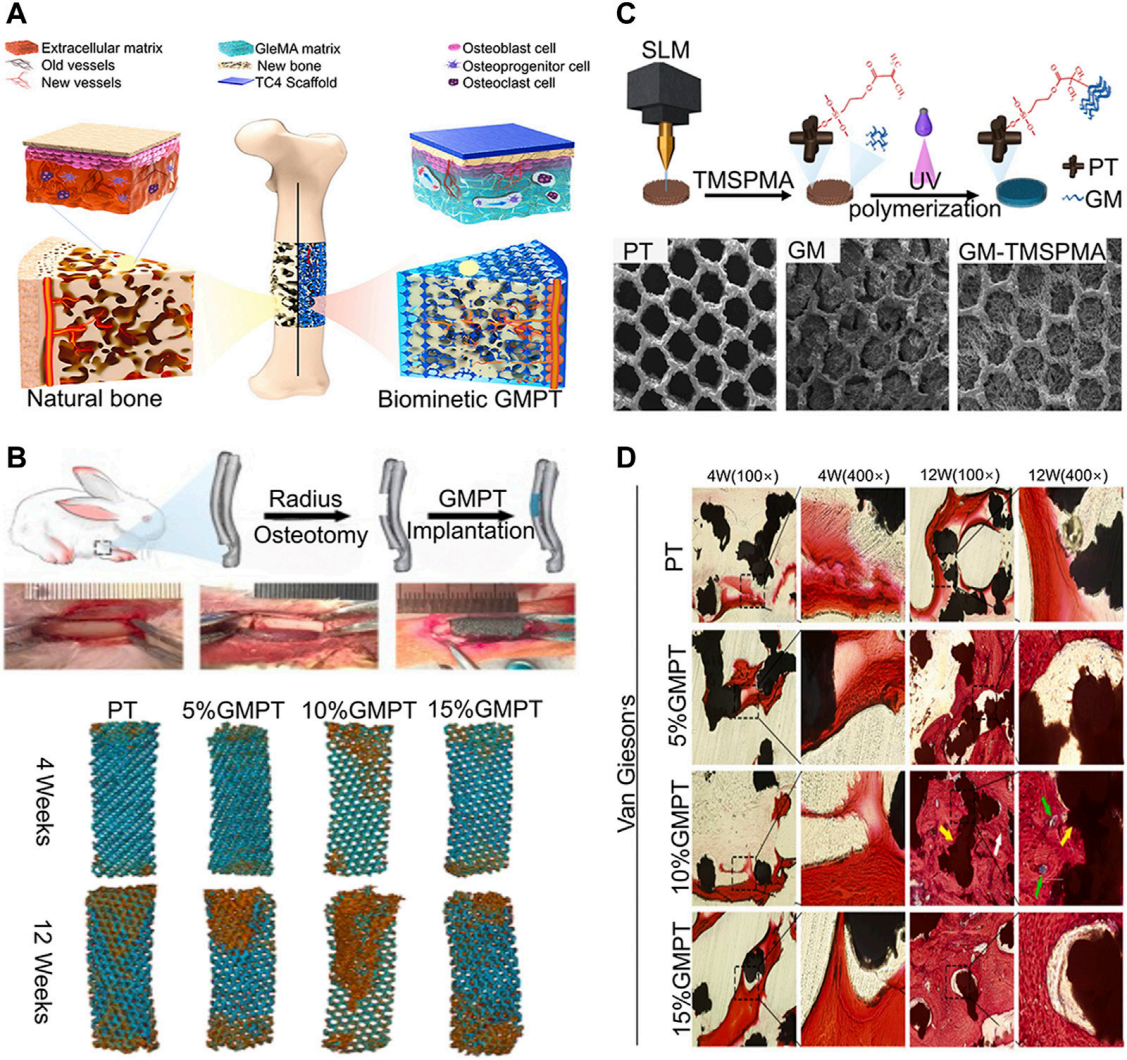
Ti6Al4V alloy/GelMA hybrid implant with dual bionic features (GMPT) for bone defect repair (Ma et al., 2021). **(A)** Schematic illustrations of the biomimetic GMPT with dual-bionic features. **(B)** *In situ* implantation of PT and GMPT implants, micro-CT 3D reconstruction of PT and GMPT in critical radius defects of rabbits. The implants in GMPT group had higher osteogenic activity than uncoated implants, and the osteogenic ability of the 10% GMPT group was the strongest. **(C)** The fabrication process and characterization of GMPT. **(D)** Histological analysis of implant samples after 4 and 12 weeks in rabbit radius defect sites. The GMPT group showed thicker and higher number of trabeculae than the PT group at both weeks 4 and 12 (yellow arrows indicate the PT implant, white arrows indicate new bone, and green arrows reveal new vessels). The 10% GMPT group showed the best osteogenesis and angiogenesis ability.

Hydrogel is a highly hydrated 3D network of interconnected polymer chains (Gibbs et al., 2016). Because of the different compositions of hydrogels, the physical and chemical properties of hydrogels vary widely. Hydrogel can be formed from natural (e.g., collagen and hypertonic acid), synthetic (e.g., polyethylene glycol), and semi-synthetic (e.g., combination of polyethylene glycol and cholesterol-containing polysaccharides) polymers (Gibbs et al., 2016). While natural polymers are typically characterized by a high degree of biocompatibility, they lack reliability and consistency due to their natural origin leading to troublesome batch-to-batch variations. By contrast, synthetic polymers are highly reproducible materials with precisely controlled chemical and physical characteristics. Unfortunately, they are classically less biocompatible than natural biopolymers either due to the material properties themselves or due to harmful residues arising from the manufacturing process which often involves cytotoxic or non-biocompatible organic solvents, starting monomers, or by-products (Utech and Boccaccini 2015). Hydrogels can absorb and retain large amounts of water (several times their dry weight) and swell, while maintaining its 3D structure, mechanical strength, and elasticity (Sosnik and Seremeta, 2017). As well being an excellent drug and cell carrier, hydrogels can be made into any shape and size according to application. Therefore, hydrogels can be completely covered in the microporous structure of 3D-printed implants (Yue et al., 2020). Hydrogels have been widely applied in the surface modification of 3D-printed titanium alloy implants. When hydrogel is used as a drug carrier, it disseminates drug molecules into surrounding tissues or cells through interconnected pores (Chyzy and Plonska-Brzezinska, 2020). For instance, Zhang W et al. (2020) developed a poloxamer 407 hydrogel loaded with simvastatin for filling porous 3D-printed titanium alloy intervertebral cages that achieved stable drug release and significantly promoted bone growth. Furthermore, the strategy of promoting bone regeneration through stem cell transplantation has become an important research topic to repair bone defects and promote bone integration. Kumar et al. (2018) developed a sodium alginate hydrogel containing preosteoblast for surface modification of 3D-printed titanium alloy. *In vitro* tests have revealed that the bioactivity of the titanium alloy implant can be significantly enhanced by adopting a hybrid system and a bioactive hydrogel. In addition, some hydrogel materials such as gelatin methacrylate (GelMA) can promote bone and angiogenesis by itself. It has a significant influence on the expression of genes associated with osteogenesis and angiogenesis across the Pi3K/Akt/mTOR pathway (Figure 4) (Ma et al., 2021).

## Functionalization of the Implant Surface

### Antibacterial Modification

Artificial joint infection is a serious complication of knee and hip replacement surgery, causing catastrophic clinical consequences (Zardi and Franceschi, 2020). With the development of implant surface modification technologies, researchers have reduced the adhesion of bacteria by loading antibacterial drugs on the implant surface and changing the surface morphology of the implant, which can effectively protect against artificial joint infection. Common antibacterial coatings include silver agents, antibiotics, polysaccharides, antimicrobial peptides (AMP), and antiparticles (Gonzalez-Henriquez et al., 2019). 3D-printed implants have a larger surface area than traditional implants, which means it has more antibacterial agents per unit volume. Accordingly, it is able to release large doses of antibacterial agents locally to obtain effective preventive and therapeutic effects (Croes et al., 2018). In view of this, 3D-printed implants with antibacterial coatings may become an effective method to prevent and treat implant infection in the future.

#### Silver Ion (Ag^+^) and Silver Nanoparticles (AgNPs) Coating

**FIGURE 5:**
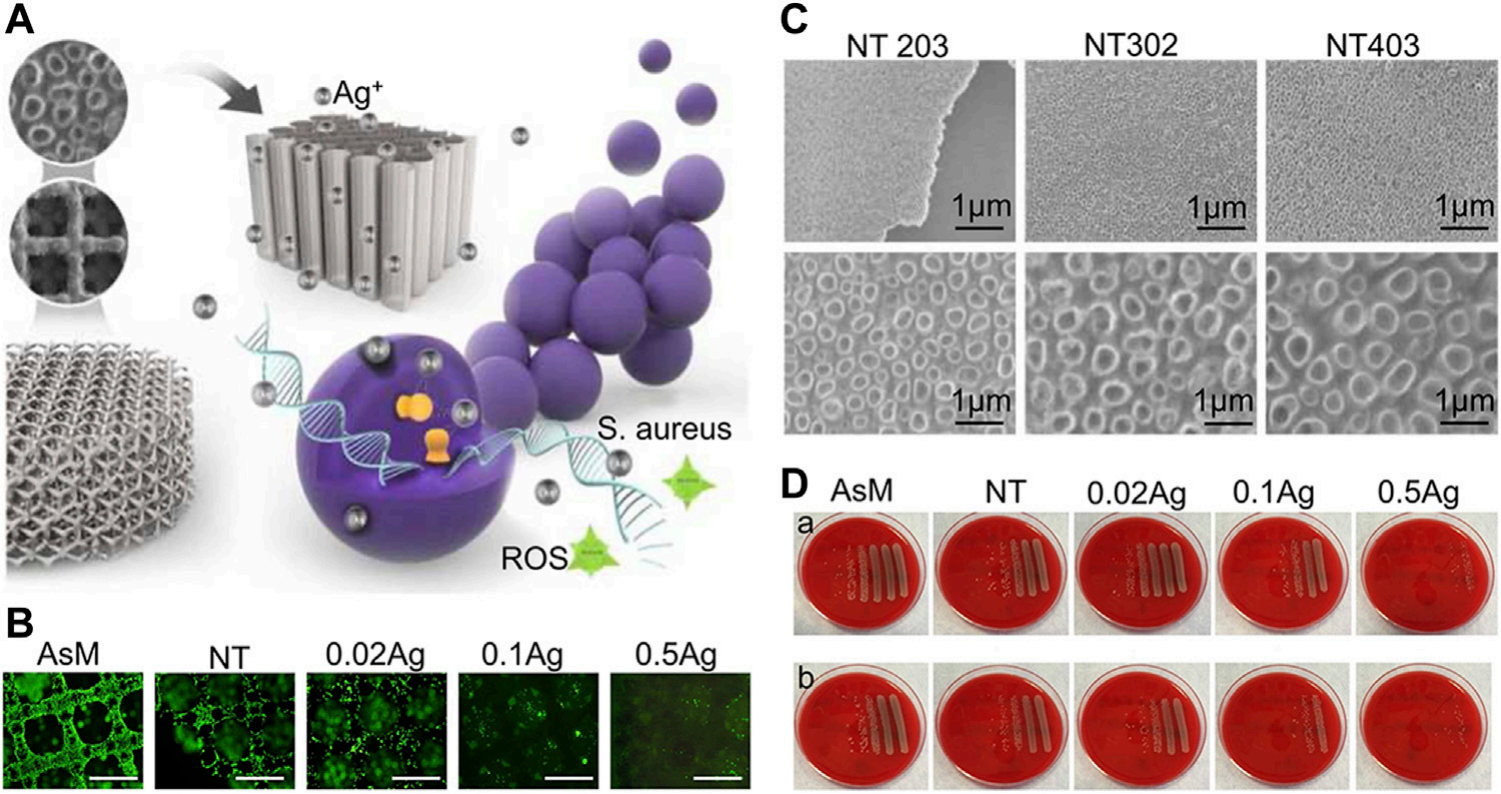
Ag^+^ coating of nanotubes prepared by anodic oxidation (Amin Yavari et al., 2016). **(A)** Schematic diagram of 3D-printed titanium alloy surface covered with nanotubes and carrying Ag^+^ to inhibit bacterial growth. **(B)** The inhibitory effect of different concentrations of Ag^+^ loaded on the implant surface on cell proliferation and adhesion. **(C)** SEM images of anodized porous titanium with the following parameters: 20 V, 3 h; 30 V, 2 h; 40 V, 3 h. **(D)** Antibacterial performance of AsM, NT, NT-0.02 Ag^+^, NT-0.1 Ag^+^, and NT-0.5 Ag^+^ against *Staphylococcus aureus* after 7 days. The first row corresponds to planktonic bacteria and the second row to adherent bacteria; the group with higher Ag^+^concentration had obvious antibacterial ability than the group with lower Ag^+^ concentration.

Ag is a highly effective antibacterial agent, specifically for drug-resistant bacteria. Ag-plated medical devices must produce an Ag^+^ concentration that is high enough to achieve the desired antibacterial effect. However, because of the cytotoxic effect of Ag^+^, it is necessary to avoid undesirable effects on cells surrounding the implant (Chernousova and Epple, 2013). When Ag is used as an antibacterial agent, it is usually made in two dosage forms: Ag^+^ and AgNPs. The antibacterial mechanisms of Ag^+^ can be divided into three categories: (1) interacting with the bacterial cell envelope, (2) interacting with molecules inside the cell (e.g., nucleic acids and enzymes), and (3) producing reactive oxygen species (ROS) (Kedziora et al., 2018). The antibacterial mechanism of AgNPs is not explicit, but the existing studies showed that AgNPs can continually release Ag^+^ to resist bacteria. In addition, AgNPs interact with bacterial cell membrane and organelles, resulting in bacterial death (Yin et al., 2020). 3D-printed implants can carry high doses of Ag agents; therefore, researchers adopted different methods to carry Ag^+^ on the surface of 3D-printed titanium alloy implants (Razzi et al., 2020). Amin Yavari et al. (2016) loaded Ag^+^ in nanotubes produced by anodic oxidation to prepare an antibacterial coating. Although large numbers of Ag^+^ show high antibacterial capability, they also cause high cytotoxicity (Figure 5). Conversely, Anish Shivaram et al. (2017) fixed Ag^+^ in nanotubes and electrolyzing Ag^+^ solution again. The Ag^+^ coating prepared by this method has a high antibacterial capacity and no evident cytotoxicity. Loaded AgNPs in carriers may further enhance the binding force between AgNPs and the implant surface, which significantly reduces the cytotoxicity of AgNPs. For instance, Jia et al. (2016) fixed AgNPs with PDA and on the micro-nano titanium alloy surface which is modified by MAO to construct an AgNPs/PDA/TiO_2_ coating. Compared with the previous modification method, this composite coating showed excellent antibacterial effect and low cytotoxicity of AgNPs. In addition, in the treatment of patients with infectious bone defect, implants are required to not only have strong antibacterial effect but also release more osteogenic substances to promote bone regeneration. Sumeneva et al. (2019) developed an AgNPs/CaP coating on 3D-printed titanium alloy implants to repair infectious bone defects. The multifunctional coating significantly inhibited *Staphylococcus aureus* and promoted bone ingrowth. Another study showed that AgNPs and SF fixed on 3D-printed titanium implants could effectively inhibit the proliferation of *S. aureus* and induce bone regeneration (Jia et al., 2019).

#### Antibiotic Coating

**FIGURE 6:**
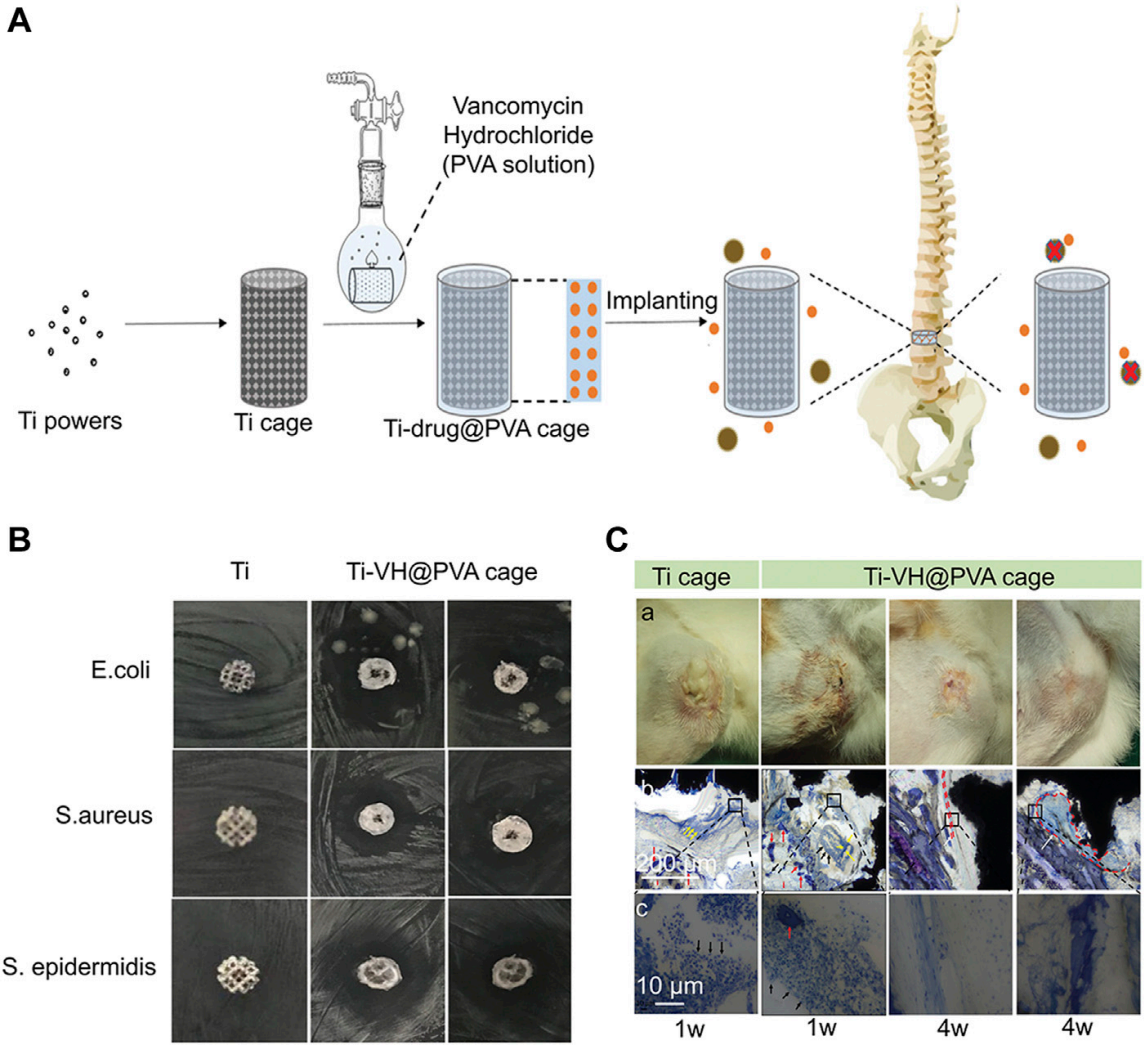
A 3D-printed titanium cage combined with a drug-releasing system for *in situ* drug release and bactericidal action (Li Y et al., 2020). **(A)** The schematic illustration of a 3D-printed titanium cage coated with PVA-vancomycin for preventing surgical site infections (Berbel, Banczek et al.) after spine surgery. **(B)** Antibacterial evaluation of Ti-VH@PVA cages *in vitro*. The obvious bacteriostatic circle was observed with regard to *Staphylococcus aureus* and *Staphylococcus epidermidis*. **(C)** Evaluation of Ti-VH@PVA cages for preventing SSIs *in vivo*. With the extension of time, the infiltration of inflammatory cells decreased significantly in Ti-VH@PVA cage. Furthermore, the thickness of the discontinuous fibrous capsule between the trabecular bone and the Ti-VH@PVA cage increased. This indicates that Ti-VH@PVA cage has a significant inhibitory effect on *Staphylococcus aureus.*

Antibiotics are the most widely used antibacterial medications, particularly in the prevention and treatment of infections after joint replacement (Miller et al., 2020; Ricciardi et al., 2020). In the post-operative stage, systemic prophylactic antibiotics are often used to preventing infection. However, the dose of systemic antibiotics is quite limited and may easily lead to bacterial drug resistance (Myers et al., 2020). Compared to systemic administration, local application of antibiotics can provide large doses of antibiotics at the surgical site, reducing the risk of bacterial drug resistance (Tan et al., 2012). In addition to the application of antibiotic containing bone cement, the antibiotic coating is a local drug delivery strategy. The antibiotic coating and 3D-printed microporous implant can further improve the local antibiotic concentration. For instance, Li X et al. (2020) developed a 3D-printed intervertebral cage covered with polyvinyl alcohol coating loaded with vancomycin that could effectively inhibit the reproduction of *Escherichia coli*, *S. epidermidis,* and *S. aureus* (Figure 6). Moreover, the combination of antibiotics and Ag^+^ may produce synergistic effects, reducing bacterial drug resistance and playing a stronger antibacterial function. Bakhshandeh et al. (2017) developed a chitosan-gelatin coating containing vancomycin and Ag^+^, which could be continuously released on the surface of the titanium alloy and was manufactured by 3D printing with high area. This chitosan-gelatin coating had better antibacterial properties than simple Ag^+^ or vancomycin coating. Aside from the simple antibacterial coating, combination of antibacterial coating and osteogenic coating may be an effective way to treat infectious bone defects. Amin Yavari et al. (2020) developed a vancomycin-hydrogel BMP-2 coating and applied it onto a porous titanium alloy surface. Results showed that the biomimetic implant efficiently inhibits bacteria within 2–3 weeks and increases the activity and mineralization of ALP on the implant surface.

### Osteointegration Modification

The intrinsic biological inertia of titanium alloy implants is a significant reason for the failure of implant surgery. Biological inertia refers to materials that can remain stable in the biological environment (Xia et al., 2020). When a biologically inert material exists in the body for a long time, it may have an exudation tissue reaction with the biological body, which can cause implant loosening and subsequent implantation failure (Han et al., 2018). In addition, bioinert materials lack osteoinduction, which may lead to the instability of bone implant binding (Wang S et al., 2018). Therefore, it has become important to increase the biological activity of titanium alloy implants, which is the focus of several ongoing research studies. Changing the surface morphology of implants (such as the surface of micro-nano structures produced by MAO) or preparing implant coatings (such as HA coatings) are two effective methods to solve this problem (Su et al., 2020; Myakinin et al., 2021). In the process of implant surface modification, these methods enhance the biological activity of the implant and promote osseointegration.

#### Calcium Phosphate Coating

CaP is a general term for a class of minerals, in which the cation is Ca^2+^ and the anions can be orthophosphate, metaphosphate, pyrophosphate, hydrogen, or hydroxide ions (Eliaz and Metoki, 2017a). When the CaP is immersed in SBF (with ion concentrations that are nearly equal to those of human blood plasma), the deposition of Ca^2+^and formation of a phosphate layer are hypothesized to be crucial steps for the initiation of the growth of bone-like apatite on biocompatible implants. The relationship between the bioactivity and solubility of the implant surface and the formation of the apatite layer in SBF is often interpreted as a first indication of its potential bioactivity *in vivo* (Surmenev et al., 2014). In general, CaP can promote the formation of new bone by inducing HA deposition on the implant surface (Eliaz and Metoki, 2017). In addition, studies found that CaP nanoparticles (CaPNPs) may have stronger ability to promote bone integration than ordinary CaP, which may be due to the increase of hydrophilicity of the implant surface caused by CaPNPs coating (Wennerberg and Albrektsson, 2009; Chudinova et al., 2019).

HA (Ca_10_(PO_4_)_6_(OH)_2_) is a form of CaP, which accounts for 70% of the bone composition (Ramesh et al., 2018). HA has superior biological activity, biocompatibility, and osteoconductivity both *in vitro* and *in vivo* (Ramesh et al., 2018). The test showed that HA coating of direct bonding strength with metal materials is weak, and it is difficult to completely cover the implant with complex shape (Huynh et al., 2019). Accordingly, HA coating is often firmly bonded to the implant surface through some modification techniques to make it play an osteogenic role by electrochemical deposition. For instance, Qin et al. (2018) prepared an HA coating on the surface of 3D-printed titanium alloy implant by anodic oxidation technology and Kodama’s alternative immersion method (AIM) that exhibited a strong bone integration ability.

#### Bioactive Glass

BAG is a type of bioactive material, involved in a variety of clinical applications in orthopedic and other hard tissue regeneration (Skallevold et al., 2019). When BAG makes contact with body fluids (BF) or simulated body fluids (SBF), it can undergo ion dissolution and glass degradation through the exchange of H^+^ in the solution and Na^+^ and Ca^2+^ from the glass network. These ions play a crucial role in osteoinduction (Rahaman et al., 2011). The most important property of BAG is induction of HA formation on the implant surface to stimulate bone growth (Skallevold et al., 2019). The composition of mesoporous BAG (MBG) is similar to that of BAG, but has a larger surface area and controllable mesoporous structure, and MBG has a stronger apatite-inducing ability (Kong C. H. et al., 2018; Lalzawmliana et al., 2020; Zhang et al., 2021). Bioactive ions such as Si^2+^ released from MBG can significantly stimulate osteogenic differentiation of osteoblasts and hBMSCs through activation of specific signaling pathways (Zhu et al., 2019). The formation of BAG coating on 3D-printed titanium alloy is a strategy to enhance osteoinduction. However, MBG coating is quickly consumed in Tris HCl buffer solution, resulting in a short time for the release of bioactive ions, so it lacks a long-term promoting effect on osteogenesis (Cerruti et al., 2005). Researchers have found that the deposition of MBG on the implant surface by anode oxidation can effectively slow down its degradation and play a biologically stimulating role (Zhao B et al., 2020). Accordingly, MBG can also be mounted on porous titanium alloy by spin coating (Ye et al., 2017). This simple method not only retains the unique mechanical structure and chemical composition of MBG but also establishes an excellent interface link with the matrix.

#### Metal Coating

Strontium (Sr) is an important osteogenic trace element which is widely used as an oral drug for the treatment of osteoporosis (Wang et al., 2020). The comparison of Sr^2+^ and other divalent ions (Mg^2+^, Ca^2+^, Ba^2+^) shows that Sr^2+^ has a more significant effect on promoting bone formation, inhibitory effect on osteoclastogenesis, immunogenicity, and fibrosis (Xu et al., 2020). The exact mechanism of Sr^2+^ in osseointegration remains unclear, but it has been proposed that Sr^2+^ acts on similar cellular targets as Ca^2+^ by activating the calcium-sensing receptor (CaSR), thus interacting with Ca-driven signaling pathways related to bone metabolism regulation (Wan et al., 2020). In addition, Sr^2+^ has high security. First, the binding ability of Sr to human plasma protein is low (∼25%), but it shows high affinity to bone tissue. Second, Sr^2+^ is adsorbed on the surface of bone minerals instead of replacing Ca^2+^ which is conducive to its rapid elimination (Martin-Del-Campo et al., 2019). Recently, it has been reported that releasing Sr^2+^ from the surface of functional implants can increase the dose of local medication, reducing side effects, and improving bone integration (Wei et al., 2020). As a result, 3D-printed titanium alloy implants with Sr^2+^ coating are well suited for osteoporosis patients requiring joint replacement surgery. Shimizu et al. and Wei et al. (2020, 2020) adopted a strategy of fixing Sr^2+^ on the implant surface with micro-nano structure to enhance the early bone binding ability by continuously releasing Sr^2+^. Moreover, to achieve long-term release of Sr^2+^ on the implant surface and obtain long-term bone induction ability, Wang S et al. (2018) developed a coating combining Sr^2+^ with zeolite to reduce the release rate of Sr^2+^ due to the zeolite cation exchange function.

Gallium ions (Ga^3+^) play an important role in orthopedic biological materials owing to its excellent antibacterial properties, inhibits osteolysis, prevents bone calcium release, and increases bone mass (Yu et al., 2020). A study showed that Ga^3+^ interacts with cellular particles and HA of bone. That means Ga^3+^ can resist bone resorption when used in patients with osteoporosis (Tharani Kumar et al., 2020). Moreover, it should be noted that Ga^3+^ also has the function of treating cancer-induced hypercalcemia and inhibiting the growth of tumor cells (Farrell et al., 2018). Therefore, 3D-printed implants with Ga^3+^ coating may be an effective means of treating bone defects caused by tumor resection in the future.

The concentration of Mg ranks fourth among all cations in the human body (Yang H et al., 2020). More than half of the Mg in the human body is stored in bone tissue in the form of biological Mg (Matsuzaki, 2006). Mg^2+^ is the most abundant cation in cells and regulates a variety of cellular functions such as cellular signal, cell growth, metabolism, and proliferation. A high concentration of Mg^2+^ can activate the calcium channels on the cell membrane (Li J et al., 2020). Furthermore, Mg is necessary for bone growth because it can inhibit osteoclast differentiation and bone resorption. A severe Mg^2+^ deficiency will lead to osteoporosis, which is characterized by reduced bone formation and increased bone resorption (Du et al., 2019). Studies showed that a small amount of dissolved Mg^2+^ is harmless in the body. Excess Mg can be metabolized by the kidneys and eventually excreted in the urine (Julmi et al., 2019). Therefore, it is important to prepare Mg^2+^ coating on 3D-printed titanium alloy implant to improve bone integration in patients with osteoporosis. Du et al. (2019) proved that the preparation of Mg^2+^ coating on the surface of 3D-printed titanium alloy implant can effectively solve the problem of poor bone integration in the osteoporosis model. Accordingly, Mg^2+^ can modify the coating with trace elements. Mg^2+^ could improve the chemical stability and mechanical resistance of CaSiO_3_ with a high degradation rate and low mechanical resistance. Tsai et al. (2019) developed a mixed coating of CaSiO_3_/Mg^2+^ and chitosan. The high bioactivity of CaSiO_3_ and Mg^2+^ significantly increased the bone induction capability of titanium alloy implants.

#### High Molecular Polymer Coating

Polycaprolactone (PCL) is a semi-crystalline aliphatic polymer commonly used to manufacture implants, drug carriers, and biodegradable packaging materials (Kiran et al., 2018). PCL has high biocompatibility, mechanical properties, processability (melting point: 60°C), and degradation absorption (Ghosal et al., 2017; Makkar et al., 2018). Moreover, PCL can maintain the porous structure of 3D-printed implants and frequently be used as a degradable carrier material for surface modification (Grau et al., 2017). For instance, Roland et al. (2015, 2016) loaded the PCL coating with vascular endothelial growth factor (VEGF), high-mobility group protein 1 (HMGB1), and other pro-angiogenic factors to stimulate the angiogenesis of porous titanium alloy implants.

Polylactide glycolic acid (PLGA) is one of the most widely used degradable materials for medical applications. PLGA has excellent mechanical properties, low immunogenicity, low toxicity, and adjustable degradation rate (Kong J. et al., 2018; Zhang H et al., 2019). Diabetes is a high-risk factor leading to implant failure. PLGA coating can alleviate diabetic condition (DC)-induced endothelial cell dysfunction and increase vascular ingrowth under DC conditions, because lactic acid (LA), the degradation product of PLGA, can stimulate angiogenesis and indirectly improve the bone formation of bone implant interface (BII) (Hu et al., 2018). Furthermore, PLGA can improve the bone integration in diabetic environment by inhibiting advanced glycation end products (AGEs) (Hu et al., 2018). In addition, as a drug carrier, PLGA materials can carry most kinds of drugs and control the release rate of drugs (Shokrolahi et al., 2019). Kwon et al. (2018) prepared PLGA coating on the 3D-printed titanium alloy implant and loaded it with ethyl 2,5-dihydroxybenzoate (E-2,5-DHB) (a drug that promotes bone formation and inhibits bone resorption). The results showed that PLGA can effectively immobilize E-2,5-DBH and ensure its steady release. Bionic implants in osteoporotic patients can effectively inhibit bone resorption around the implant and promote bone scarring.

Dopamine (DA) is used as the sole adhesive protein of mussel foot-5 (MEFP-5) and can be associated with various metal ions through its dispersed alcohol groups (Li et al., 2016). Inspired by the natural mussel adhesion phenomenon, DA could self-polymerize to form PDA coating under mild conditions through a simple experimental process (Huang et al., 2016). PDA is used in implant coating for different purposes, such as antibacterial treatment, protein binding, cell culture, and medication administration (Perikamana et al., 2015). The catecholamine-rich PDA coating was found to contribute to the formation of HA on various implanting systems (Kaushik et al., 2020). As is known, PDA is an excellent drug carrier, which can fix AgNPs, HA, vancomycin, magnetic Fe_3_O_4_ oxide, and other drugs on the surface of the implant to achieve its biological functions (Li Y et al., 2015; Jia et al., 2016; Zhang T et al., 2018; Lingpeng Kong et al., 2020).

#### Organic and Bioactive Coating

Protein coatings have been key for resolving the poor osseointegration of implants over the past few years. Proteins as implant coating materials can significantly increase the binding force of bone and implant integration, because the surface of the implant covered by the protein is highly recognized as a “host type” tissue and the addition of specific biological clues can change the response of the bone tissue around the implant. Consequently, osteoblasts can be attached to the implant surface more quickly to stimulate bone regeneration (Jurczak et al., 2020). Common protein coatings include growth factors (BMP-2, BMP-7, VEGF), type I collagen (COL I), and albumin.

BMP-2 is an obstetric cytokine in the transforming growth factor-β (TGF-β) family and is currently the most commonly used protein-based bone transplant substitute (Nguyen et al., 2017). BMP-2 plays a major role in embryonic development, bone remodeling, and homeostasis in adulthood. BMP-2 can activate osteogenic genes such as Run-Related transcription factor 2 (*RUNX2*) to promote bone growth (Halloran et al., 2020). Preclinical and clinical studies showed that BMP-2 can be applied for the treatment of a variety of orthopedic diseases such as bone defects, nonunion, spinal fusion, osteoporosis, and root canal surgery (Chen et al., 2004). The incorporation of BMP-2 on implant surfaces appeared as a new strategy aimed at improving osteogenic activity and osseointegration of implant surfaces. A test revealed that BMP-2 can be released steadily on the 3D-printed titanium alloy surface which treated by MAO and the osteoinduction of implants is significantly enhanced (Teng et al., 2019). In addition, it is worth mentioning that BMP-2 can be covalently coated by 3D-printed titanium alloy by ion-assisted plasma polymerization technology to play its osteogenic role (Croes et al., 2020).

Parathyroid hormone (PTH) has the potential to enhance bone regeneration of bone abnormalities (Wojda and Donahue, 2018). Osteostatine is the C-terminal N-terminal 107–111 sequence of PTH. The short length and amino acid composition of this pentapeptide provides stability, and osteostatine has stimulated osteoblast activity and inhibited osteoclast activity. Studies have shown that osteostatine coating on the 3D-printed implants can be effective for bone regeneration (van der Stok et al., 2015).

**FIGURE 7:**
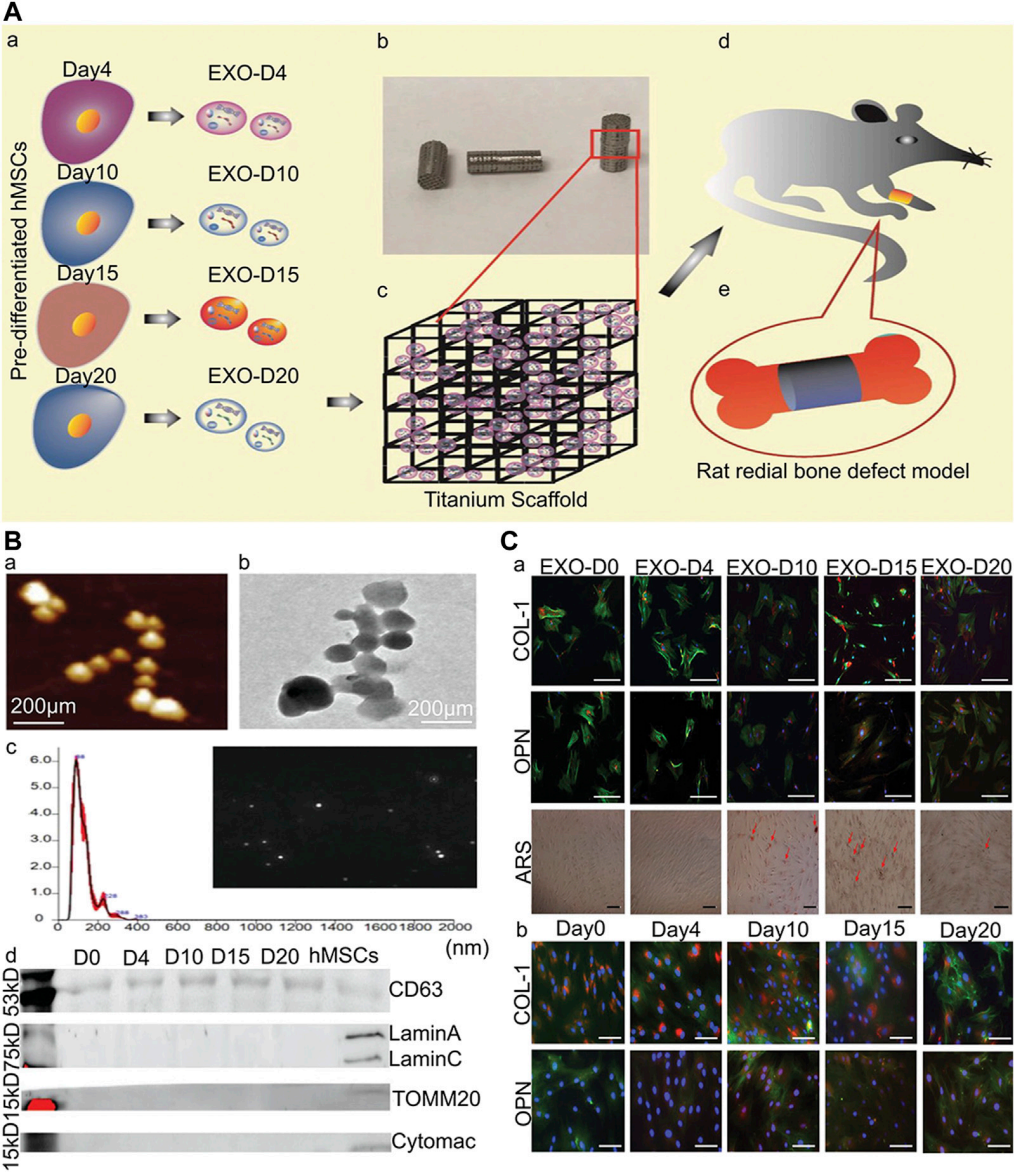
Osteogenic exosomes induce osteogenic differentiation (Zhai et al., 2020). **(A)** The schematic illustration of cell-free bone tissue regeneration by the stem cell-derived exosomes. **(B)** The characterization of the stem cell-derived exosomes. a) AFM and b) TEM showing the size and morphology of the exosomes derived from hMSCs. Scale bar: 200 nm. c) The size and concentration of the hMSCs-derived exosomes by the NanosightNS300. The inset is an image showing the snapshot of video tracking. d) The western blot analysis of the exosome derived from the pre-differentiated hMSCs and hMSCs. **(C)** Osteogenic differentiation of hMSCs by the osteogenic exosomes a) Immunofluorescence staining of osteogenic markers (COL-1, OPN) in hMSCs induced by osteogenic exosomes. b) Immunofluorescence staining of osteogenic markers (COL-1, OPN) in hMSCs induced by osteogenic medium. There was no significant difference between osteogenic exosomes treatment and osteogenic medium treatment, which proved the osteogenic induction function of osteogenic exosomes.

Introducing organic molecules with functional fragments in the lining is a new strategy to promote osteogenesis. RGD is an artificial peptide that has a specific bio-active sequence. It exists in most ECM proteins including fibronectin, vascular junction protein, and laminin (Hersel et al., 2003). RGD stimulates bone integration by increasing the proliferation and adhesion of osteoblasts on the implant surface (Elmengaard et al., 2005). One study reported that the hybrid molecular coating obtained by chemical crosslinking of amino acid bisphosphonate and linear tripeptide RGD combines, which has a strong ability to stimulate bone integration (Parfenova et al., 2020). Furthermore, another study showed that identifying the exons of hMSC osteogenic differentiation and binding them to the implant surface can achieve acellular bone regeneration and the bone tissue regeneration efficiency of acellular coated implants was equivalent to hMSCs-implanted acellular implants (Figure 7) (Zhai et al., 2020).

COL I is the most abundant bone protein (representing 85% of organic constituents) (Sartori et al., 2015). The interaction between cells and collagen plays an important role in regulating the differentiation of bone marrow in the osteogenic pathway (Sartori et al., 2015). Studies have shown that COL I has the capacity to recruit osteoblasts and accelerate mineralization, and enhance bone regeneration (Naomi et al., 2021). COL I of calf skin or mouse tail has been extensively used as a biological coating material for titanium alloy implants. Veronesi et al. (2021) were successful in increasing the bioactivity of implants by binding type I collagen on the surface of a 3D-printed titanium alloy.

Decellularized extracellular matrix (dECM) is a tissue ECM isolated from primitive cells (Yao et al., 2019). With proper detection methods dECM can be easily obtained from tissues/organs of different species (Kim Y. S. et al., 2019). dECM can simulate the structure and components of the natural ECM and offer a good growing environment for cells (Yao et al., 2019). One study showed that dECM coating on the 3D-printed titanium alloy implants can increase cellular proliferation and adhesion to promote bone ingrowth (Kumar et al., 2016).

Quercetin is a class of natural flavonoids, widely found in flowers, leaves, and fruits of various plants, and has anti-cancer, anti-fibrotic, anti-inflammatory, and antioxidant effects (Tang et al., 2019). Llopis-Grimalt et al., (2020) prepared a quercetin coating on the porous 3D-printed titanium alloy. *In vivo* test results showed that the quercetin coating had excellent antibacterial capacity and osteogenic induction capacity.

### Mechanical Properties Modification

#### Wear Resistance Modification

**FIGURE 8:**
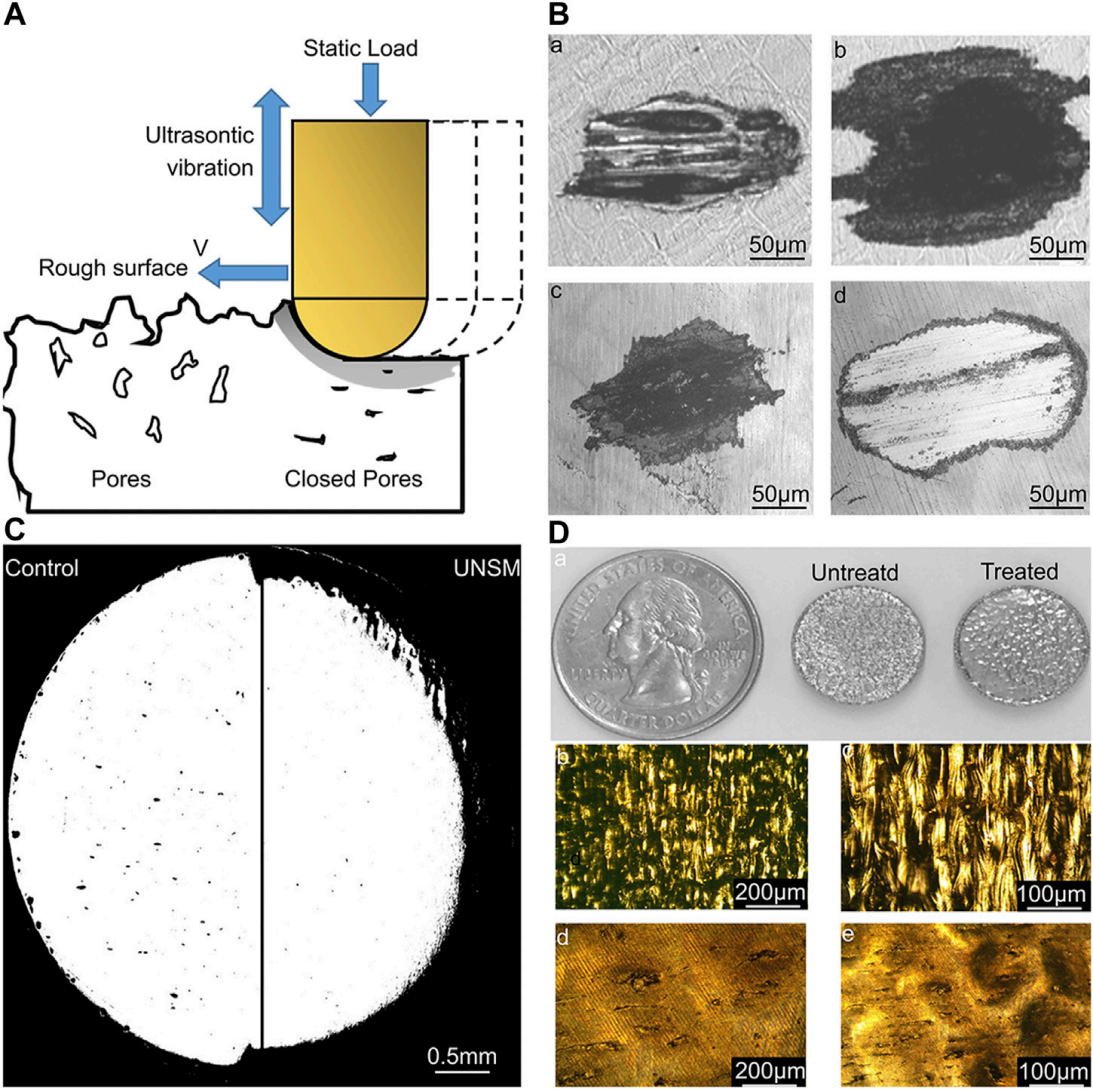
Effect of UNSM treatment on 3D-printed NiTi alloy surface (Ma et al., 2017). **(A)** Schematic of the UNSM processing showing its effect on surface finish, subsurface porosity, and surface hardening. **(B)** Wear scars of untreated (a and b) samples and UNSM-treated (c and d) samples at 6,000 (a and c) and 24,000 (b and d) cycles. **(C)** Porosity distribution on the untreated surface and UNSM-treated surface. **(D)** a) Appearance of the AM NiTi samples before and after UNSM treatment; b) and c) show the optical images of the non-treated sample. d) and e) show the optical images of the UNSM-treated sample. The treated samples have better wear resistance and lower porosity than untreated samples.

Aseptic loosening of the implant is a crucial cause of implantation failure (Apostu et al., 2018). The wear and adhesion of polyethylene, metal, and bone cement in the body may produce debris particles, which cause aseptic inflammatory reactions in the tissues surrounding the operation, leading to bone resorption and implant loosening (Bauer and Schils 1999). Therefore, enhancing the wear resistance of the implant and reducing the generation of wear particles can diminish the incidence rate of implant operation failure. Lately, discontinuous fiber-reinforced titanium-based composite coatings synthesized *in situ* by high-energy lasers have been extensively used. In comparison to monolithic coatings, these coatings have excellent mechanical properties, such as high toughness and excellent mechanical strength (Das et al., 2014). In addition, changing the surface morphology of an implant is also a way to improve its wear resistance. A study reported that the surface modification of NiTi alloy by UNSM, significantly enhanced the wear as well as corrosion resistance of the implant without changing the chemical properties of the implant surface (Figure 8) (Ma et al., 2017).

#### Fatigue Strength Modification

Although 3D printed titanium alloy stents have many advantages such as personalized customization and matching the elastic modulus of human bones, the fatigue strength of 3D printed titanium alloys is considerably weaker than traditional titanium implants (Soyama and Takeo, 2020). In addition, surface modification technologies (MAO, anodizing, plasma spraying, sand blasting) on the surface of titanium alloy will reduce the fatigue strength of implants (Yang Y. W et al., 2020). To enhance the service life of the implant, it is necessary to increase the fatigue strength of the implant. The fatigue strength is closely related to the porosity and surface morphology of implants (Kelly et al., 2019). In addition to using high-strength implant materials, surface modification may be a strategy to enhance their fatigue strength. Studies have proven that shot peening on the surface of 3D-printed titanium alloys can effectively improve the fatigue strength of implants (Remigiusz ˙Zebrowski et al., 2019; Soyama and Takeo, 2020). In addition, the TiO_2_ nanotube coating on the surface of titanium alloys by hydrothermal treatment can also effectively improve the fatigue strength of the titanium alloy (Yang Y. W et al., 2020). At present, there are few studies on surface modification to improve the fatigue strength of 3D-printed titanium alloy. Further improving the fatigue strength of 3D-printed implants may depend on the design of the implant materials and shapes.

#### Corrosion and Oxidation Resistance Modification

The corrosion resistance of the implant is an important property of the implant, and the level of corrosion resistance determines the biocompatibility of the materials. The lower the corrosion resistance of the implant, the higher the toxicity of the ions released into the body (Asri et al., 2017). The reason why titanium alloy has a great corrosion resistance is that the passive oxide film will be formed on the surface of the titanium alloy after contact with oxygen (Berbel et al., 2019). When the artificial joint is implanted *in vivo*, the TiO_2_ on the surface of the implant may be corroded by the body fluid environment, resulting in the reduction of the corrosion ability of the implant (Rodrigues et al., 2009). In order to increase the service life of the implant and decrease the risk of postoperative adverse reactions, it is necessary to prepare a corrosion-resistant coating. A study showed that chemical polishing of 3D-printed titanium alloy materials can significantly enhance the thickness of the TiO_2_ layer of its surface, thus increasing the corrosion resistance of titanium alloys (Zhang Y et al., 2019). Furthermore, the corrosion resistance of titanium alloy can also be improved by MAO treatment (Rafieerad et al., 2015). This may be attributed to the fact that MAO can improve the bonding strength between the coating and titanium alloy and enhance the strength of crystal structure in the coating.

## Expectation

3D-printed titanium alloy implants have attracted much attention in orthopedics due to excellent biocompatibility, elastic modulus similar to natural bone, and 3D shape to fit complex bone defects. Implant surface modification technique is an important force that promotes the first generation of implants (biologically inert with good mechanical properties) towards implants with excellent osteoconductivity, osteoinduction, bone healing, and remarkable anti-inflammatory infection capability. This article reviewed various surface modification techniques in the 3D-printed titanium alloy and its improvement in the function of implants. However, further research is essential before these techniques can be clinically applied. Therefore, we provide some future views on the development of 3D-printed titanium alloy surface modifications, which can be used as the basis for further improvement.

### Implant Surface Modification to Regulate Pathological Conditions

Most current implant surface modification techniques are aimed at the functionalization of implants under normal physiological conditions. However, many patients who need joint replacement are elderly. Senior patients who suffer from underlying diseases (hypertension, diabetes, coronary heart disease, osteoporosis, tumors) probability is relatively high. Although there are some studies on diabetes and 3D-printed titanium alloy implants with respect to osteoporosis, these studies are only preliminary investigations. In future, the mechanism of bone integration under pathological conditions needs to be extensively studied according to various diseases. In addition, further research on 3D-printed implant coating related to cancers and rheumatic immune diseases needs to be designed. Moreover, it also remains to be seen whether the implant coating for the above pathological conditions, which has been applied on the surface of ordinary implants, can play a role in 3D-printed implants. The combination of degradable 3D implants (PCL, PLGA) with hydrogels can provide an answer to these problems. Dual degradable materials can increase the amount and type of drug loading and determine the time sequence of drug release. The order of drug release can be determined according to the substances required by the new bone tissue.

### Preparing Bioactive Material Coatings With Strong Adhesion

The application of bioactive substances as coating materials has recently become a research hotspot. However, because proteins cannot withstand extreme environments such as high temperature and high pressure, researchers often use dip coating to prepare coatings. This leads to weak adhesion between the coating and the implant. Although improved vapor deposition methods such as PCVD and ICVD can greatly reduce the temperature required for deposition, the preparation of biological coatings has not been attempted. Of course, these coating preparation methods have great potential in the preparation of strong-binding biomaterial coatings. In addition, changing the surface roughness of the implant is another way to enhance adhesion between coatings and implants materials. Studies have reported that the micro-nano surface prepared by MAO or anodic oxidation can enhance the adhesion between implant surface and coating, but optimum electrolytic parameters need to be explored further.

## Conclusion

In this review, various types of 3D implant surface modification techniques including their principles, processes, advantages, disadvantages, and applications are discussed. In addition, this article summarizes the various types of functional modifications, as well as improvements in osteogenesis, antibacterial, wear resistance, corrosion resistance, and oxidation resistance caused by different coating materials and modification methods. Based on these findings, future research should determine the best parameters suited for surface modification techniques and the most suitable coating materials, as these are significant factors for optimizing orthopedic implants.
